# Near Real-Time Volumetric Estimates Using Unmanned Aerial Platforms Equipped with Depth and Tracking Sensors

**DOI:** 10.3390/s22239462

**Published:** 2022-12-03

**Authors:** Donato Amitrano, Luca Cicala, Giovanni Cuciniello, Marco De Mizio, Mariana Poderico, Francesco Tufano

**Affiliations:** Italian Aerospace Research Centre, Via Maiorise snc, 81043 Capua, Italy

**Keywords:** close-range remote sensing, drones, near real-time three-dimensional scene reconstruction, volume estimation, Kalman filtering, Intel RealSense

## Abstract

Volume estimation of specific objects via close-range remote sensing is a complex task requiring expensive hardware and/or significant computational burden, often discouraging users potentially interested in the technology. This paper presents an innovative system for cost-effective near real-time volume estimation based on a custom platform equipped with depth and tracking cameras. Its performance has been tested in different application-oriented scenarios and compared against measurements and state-of-the-art photogrammetry. The comparison showed that the developed architecture is able to provide estimates fully comparable with the benchmark, resulting in a quick, reliable and cost-effective solution to the problem of volumetric estimates within the functioning range of the exploited sensors.

## 1. Introduction

Measuring the volume of specific objects within a scene is a complex task requiring sensors or data processing techniques able to generate dense point clouds. This is usually done by means of LIDAR technology [1] or the fusion of several pictures acquired from different view angles through the structure from motion (SfM) algorithms [2]. Despite the reliability of these approaches, some weaknesses exist, such as the high computational burden required to process point clouds or the expertise requested by operators to effectively exploit these tools. This prevents the spreading of the technology in many sectors which could benefit from quick and reliable volume estimates, such as logistics [3], earthworks [4], or agriculture [5].

The architecture presented in this paper has been developed under the aegis of the research and development project “Crowd for the environment,” co-funded by the Italian Ministry of Research. Its goal was the development of a progressive and multi-layer monitoring system able to provide decision-makers with continuously updated environmental risk maps through the integration of heterogeneous information, from crowdsourced data to satellite acquisitions.

One of the targets of interest in this context is micro-dumps, which are one of the major environmental hazards in many urban and suburban areas throughout the world [6]. Their typical dimension makes it difficult to monitor from space [7], especially concerning the occupied volume, which is an essential parameter for the evaluation of the dangerousness of a site. However, the aforementioned limitations represent significant obstacles in the typical application domains of the technology because on-field operators are typically requested for a quick (and even rough) evaluation of the site to establish an intervention priority scale. 

This paper presents a new architecture for real-time volume estimates exploiting state-of-the-art hardware and innovative software components. The hardware design includes a custom drone equipped with navigation and optical sensors able to deliver the information needed for the direct positioning of depth frames in three-dimensional world reference systems. They are a PixHawk autopilot and two Intel RealSense cameras, i.e., a tracking camera and a depth camera [8].

The core of the software component is constituted by an innovative algorithm able to coherently place the acquired frames in a three-dimensional reference frame. Then, the reconstructed scene is automatically segmented for the extraction of its objects and volumetric estimates. The whole process has been designed under a near real-time constraint of 2 min for on-field operations.

To date, this is the first work proposing the exploitation of an aerial platform equipped with RealSense cameras for mapping purposes. Recently, some authors proposed a similar setup for the autonomous navigation of ground robots and/or recognition tasks. As an example, Reference [9] presented an approach combining the Intel RealSense T265 tracking camera and the Intel RealSense D435 depth camera as a navigation system for a hexapod walking robot. Reference [10] used the two sensors for person localization in an industrial site. Some works exploited only the depth camera for drone localization [11], skeletal hand tracking [12] and real-time three-dimensional volumetric reconstructions [13].

The paper is organized as follows. In Section 2, the developed hardware and software are discussed in detail. Experimental results are provided in Section 3 and discussed in Section 4. Conclusions are drawn at the end of the work.

## 2. Materials and Methods

### 2.1. The Platform

**Figure 1:**
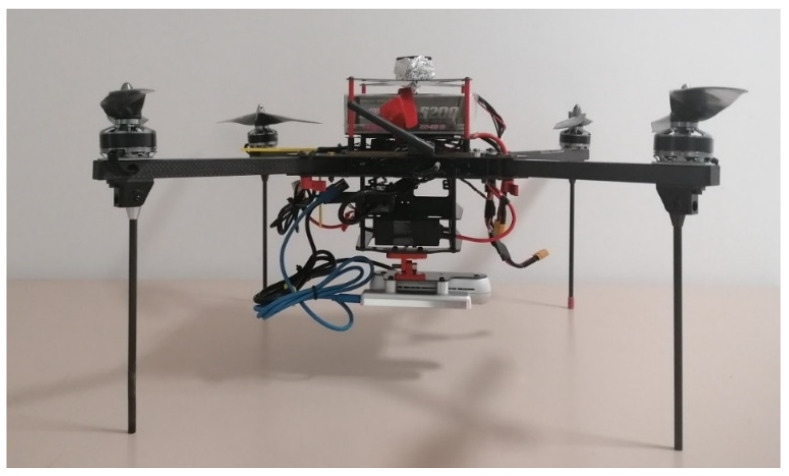
The exploited aerial platform.

**Figure 2:**
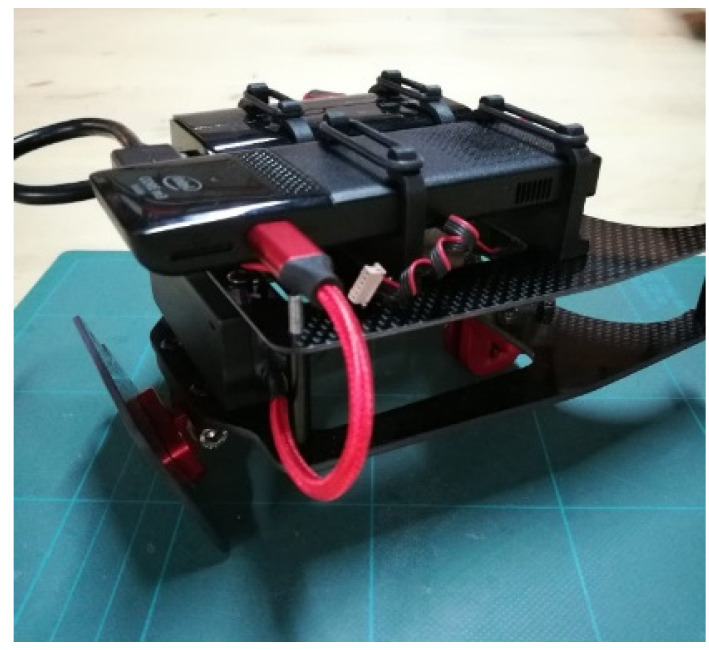
Customized mechanical support housing the onboard CPU and the USB hub.

The exploited unmanned aerial platform is a customized Foxtech Hover 1 Quadcopter (Figure 1). The chassis of the platform has been modified to house, in addition to the original components, an onboard computer, USB hub, a new power management system and mechanical support to install the payload (Figure 2). The payload bracket is bonded to the carbon fibre chassis with a hinge allowing for different camera orientations. 

The customization caused the quadcopter weight to increase from 2.4 kg to 3.1 kg. Therefore, to guarantee the safety of the fly, the original battery and propellers have been changed to more performing ones. The original battery was a 9500 mAh Lion battery with a 3C discharge capacity. It has been substituted with a 5200 mAh LiPo battery with a 35–55 C discharge capacity. The new propellers are the CAM-Carb.Light 14 × 5 L. They allow for improved trust performance with respect to original ones at the expense of reduced autonomy from about 40 min to 15 min. However, this is not expected to impact operations, as the envisioned applications do not require long surveys. 

The Intel Pc Stick Stk2mv64cc is used as an onboard computer. It is connected with the payload (see Section 2.2) and with autopilot through a USB 3.0 and a USB 2.0 connection, respectively. A specific software application has been developed in order to acquire RGB images, depth images, inertial measurement unit (IMU) data and positioning information from the payload and the autopilot. The acquired data are recorded together with their own timestamp allowing for the synchronization necessary for subsequent processing.

### 2.2. The Payload

The payload consists of three subsystems, i.e., a depth camera, a tracking camera, and a navigation system. All of them are commercial components. Cameras are produced by Intel (RealSense product line). The navigation system is a PixHawk autopilot.

The depth camera is the main acquisition system for the target application. It is composed of an integrated group of sensors, i.e., two cameras working in the near-infrared (IR) spectral band mounted in a stereo configuration, an IR illuminator that projects a structured light pattern, and an RGB camera. The camera is equipped with an IMU based on micro-electro-mechanical systems (MEMS) technology. The purpose of such a vision system is to deliver a 3D point cloud of the observed scene, calculated by processing a depth map acquired in the IR spectrum using structured light. The additional geometrically calibrated RGB camera allows for colorizing each point based on its visible spectrum response. According to the vendor, the depth accuracy of such a vision system is higher than 2% at 4 m of distance from the target, while the declared functioning range is 7 m. 

The precise knowledge of the camera position needed to join the different snapshots reconstructing an extended scene requires the use of a second vision system to enhance the positioning performance, i.e., the tracking camera Intel Real Sense T265.

This sensor is composed of two cameras working in the near-infrared spectral band mounted in a stereo configuration, an IR projector, and an IMU equipped with accelerometers and gyroscopes. This configuration allows for delivering an estimate of the pose of the camera with centimetric accuracy. In the proposed configuration, the tracking camera is mounted with the depth camera in a fixed and stable mechanical configuration. An issue of this type of vision system is the position drift within non-closed trajectories. On the other hand, the manufacturer reports a position error of less than 1% on closed trajectories.

The two cameras are jointly exploited to produce geocoded point clouds ready to be collocated within the three-dimensional space. Due to reduced computational burden with respect to standard photogrammetry, onboard processing and real-time applications are enabled. The positioning performance is improved thanks to the navigation system of the platform flight controller, which, beyond the IMU, is equipped with a magnetometer and a Global Navigation System Receiver (GNSS). The redundancy of the measures and the complementarity with respect to those of the two vision systems allows for obtaining a more accurate and robust pose estimate against drift effects via Kalman filtering. This will be discussed in detail in Section 3.4.

### 2.3. Real-Time Positioning of Point Clouds and Volumetric Estimates

**Figure 3:**
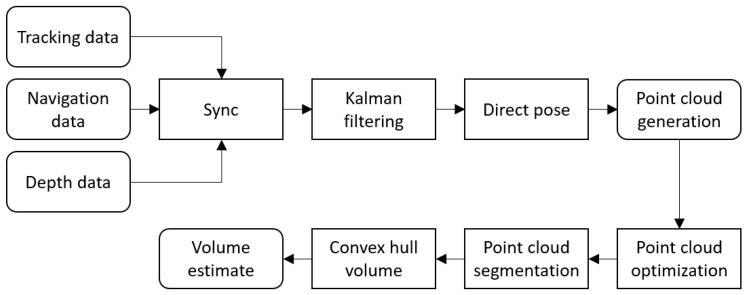
Proposed workflow.

The adopted workflow is sketched in Figure 3. In this diagram, blocks with rounded corners indicate data (input or generated). The rectangles stand for processing blocks.

The starting points are the streams the navigation system provides, the depth camera, and the tracking sensor. They are synced at runtime in order to allow direct pose of pictures up to a lag of 10 milliseconds. 

After synchronization, data are re-projected in a common world reference system through coordinate transformation. Given the position of the generic point of the cloud $x_{d}$ provided by the depth camera, the transformation to world coordinate is defined by the relation

(1) $$x_{w}=T_{t2w}T_{d2t}x_{d}\;,$$

where $T_{d2t}$ and $T_{t2w}$ identify the transformation from the depth camera frame to the tracking camera frame and from the tracking camera frame to the world frame, respectively. 

The rotation matrix $T_{t2w}$ defining the relative orientation between the pose frame and the world frame is provided by a sensor fusion system, as discussed in Section 2.4. The transformation from the depth frame to the tracking frame, i.e., the matrix $T_{d2t}$ is known from camera mounting. It is defined by the three-dimensional translation vector and rotation matrix, determining the offset and the relative orientation between the depth and pose frame. Mathematically, given the in-depth frame, the homogeneous transformation to the pose frame is defined by

(2) $$x_{t}=\left[\begin{array}{ccc} r_{11} & r_{12} & r_{13}\\ r_{21} & r_{22} & r_{33}\\ r_{31} & r_{32} & r_{33} \end{array}\right]\;x_{d}+\left[\begin{array}{c} t_{x}\\ t_{y}\\ t_{z} \end{array}\right].$$

Using the bracket drawing provided by the producer for replication, the transformation (2) is known [14]. 

According to Figure 3, the pose of the tracking camera in a geographical north east down (NED) reference system is generated through the fusion of the tracking camera pose and IMU data, the depth camera IMU data, and the navigation data provided by the autopilot via Kalman filtering (KF). Based on the filtered position, the depth frames made available by the depth camera (opportunely decimated to reduce the computational burden) are directly placed within the world frame and joined into the final (sparse) scene point cloud. A spatial alignment between couples of temporally adjacent point cloud frames is performed with the iterative closest point (ICP) registration algorithm [15] to further improve the precision of the positioning of the point clouds, thus recovering residual misalignment errors.

Segmentation involves two tasks, i.e., identifying the ground plane and the objects populating the scene. The first objective is reached using the maximum likelihood estimation sample consensus (MLESAC) algorithm [16]. The overground scene is then treated with three-dimensional automatic segmentation to isolate single objects. 

This is done by selecting a random seed and a control volume for the search of neighbor points. The initial seed grows as the moving control volume contains points to be aggregated to form an object. When the control volume is empty, it means that there are no more points to be aggregated to the current object. Thus, another seed is selected, and the process repeats until no more points belonging to the clouds are assigned to a new object [17].

Finally, the volume of each identified object is estimated as the volume of the three-dimensional shape identified by the points representative of each scene object [18].

### 2.4. Positioning Improvement via Kalman Filtering

As mentioned above, the availability of a global navigation satellite system (GNSS) receiver and redundant pose measurements provided by the navigation systems of the different sensors equipping the platform opens to integration/fusion of such measurements to obtain a more accurate and robust pose estimation against drift effects. Similar approaches have been previously proposed in [19,20]. In these works, data from the navigation system of the flight controller have been fused with those provided by a monocular camera, operating, respectively, at close distance and high altitude from the ground plane in order to estimate linear and angular velocities of the platform.

To this end, sensor extrinsic cross-calibration is necessary, being the relative orientation between the cameras’ reference systems and the autopilot unknown. This information can be retrieved by calculating the reciprocal orientation of the available inertial measurement units (IMUs) by analyzing the phase displacements between their acceleration signals.

First, a long time series of acceleration measurements are acquired during consecutive movements of the drone. The pose between two consecutive movements is held for a few seconds before moving the platform to another position. In such a way, a stable signal is produced with sharp acceleration peaks along a well-defined direction [21].

The second step of the calibration procedure consists of calculating the acceleration module for all the sequences. If necessary, signal lags must be opportunely compensated. 

Finally, the rotation matrix representing the rigid body transformation from the input reference system, i.e., the tracking camera one, to output world coordinates is calculated via singular value decomposition [22].

This process avoids implementing a model for estimating the extrinsic parameters in the sensor fusion algorithm. As a consequence, it is not necessary to start the flight in advance to allow the KF to converge to a solution for the calibration parameters.

**Figure 4:**
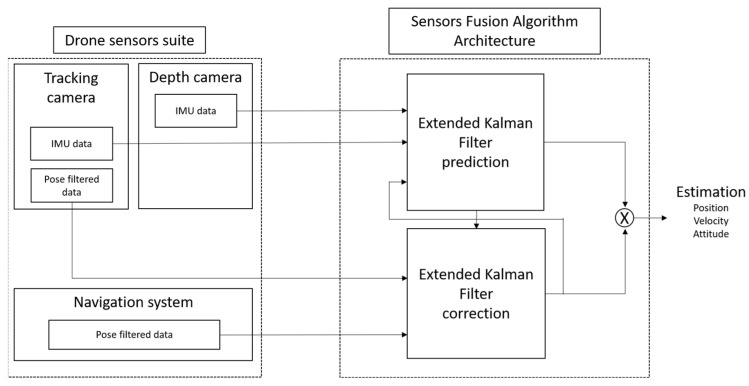
Sensor fusion algorithm architecture.

After cross-calibration, sensor fusion can be implemented via extended Kalman filtering (EKF) [23]. The filter architecture is depicted in Figure 4. It combines statistical mathematics with linear system theory in a recursive algorithm that computes maximum likelihood estimates of a system state. In particular, this filtering technique is very effective in reaching a reliable estimate of a time-varying process by updating current state estimates using past measurements and covariance estimates without their reprocessing at each iteration.

As shown in Figure 4, the filter input is constituted by the IMU measurements of the payload, which are exploited for the filter prediction step in order to obtain the state vector estimate. The correction step has been implemented considering the navigation system filtered data and tracking camera filtered data. The tracking camera data are exploited to correct the linear velocity estimation, while GNSS is used in order to avoid position drifts on open trajectories and to geocode the point clouds.

## 3. Experimental Results

This Section presents the results of several experiments implemented in different environments and involving different targets. Different applications, such as logistics, agriculture, earthworks, and environmental monitoring, have been analyzed. 

The first experiment was implemented in an indoor GNSS-denied environment with the objective of testing the system’s capability in scene reconstruction mapping using a rather dense grid. The experiment involves targets of known geometry and volume. 

The second experiment was performed in an outdoor environment with airborne instrumentation for volumetric estimations, thus accepting lower-quality mapping due to the usage of sparse clouds. Again, targets of known geometry and volume have been surveyed. 

The third experiment was implemented in a construction site and concerned the survey of a gravel pile. The fourth and last experiment consisted of the survey of a micro-dump.

### 3.1. Experiment 1—Indoor GPS Denied Environment

**Figure 5:**
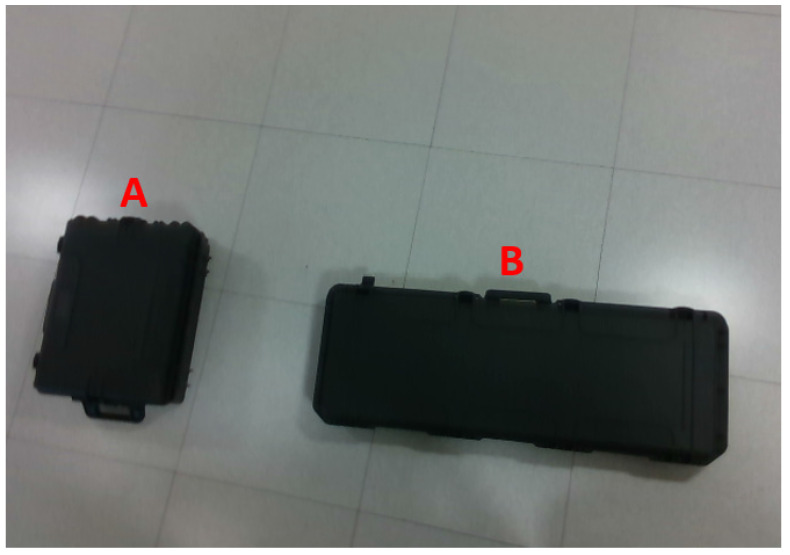
Target objects for indoor 3D reconstruction and volume estimation.

The first experiment was implemented in an indoor environment. Cameras were mounted on a cart equipped with a boom with approximative nadir pointing in order to simulate possible oscillations of an aerial platform. The target objects were two boxes placed on the floor, as shown in Figure 5. Their measured volume is $V_{A}=0.050\;m^{3}$ and *V_b_* = 0.086 m^3^. 

**Figure 6:**
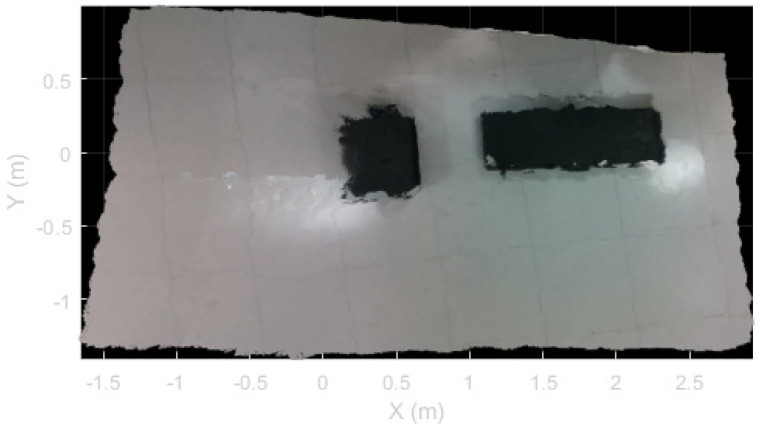
Three-dimensional scene reconstruction obtained via direct pose of pictures.

The 3D scene reconstruction obtained via direct pose of depth frames is shown in Figure 6. The reconstruction grid was set to 1 cm to account for the objects’ size. Quantitative data about the estimated volumes and the acquisition are reported in Table 1.

Qualitatively, the scene reconstruction obtained through the direct pose method is of good quality and characterized by small distortions of the imaged objects. Working in an indoor GNSS-denied environment, pose data have been extracted from the tracking camera. Quantitatively, volumetric estimations are comparable with those obtained using Agisoft Metashape through state-of-the-art SfM photogrammetry. However, the reconstruction grid is about three times higher in the direct pose case. Both estimations are in line with measurements with a good degree of approximation. In particular, for the direct pose experiment, the percentual error on the volumetric estimation is 4% for box A and 10.5% for box B. Using Agisoft Metashape, the registered errors are in the order of 23.1% for box A and 26.5% for the box B.

### 3.2. Experiment 2—Simple Targets in Outdoor Environment

**Figure 7:**
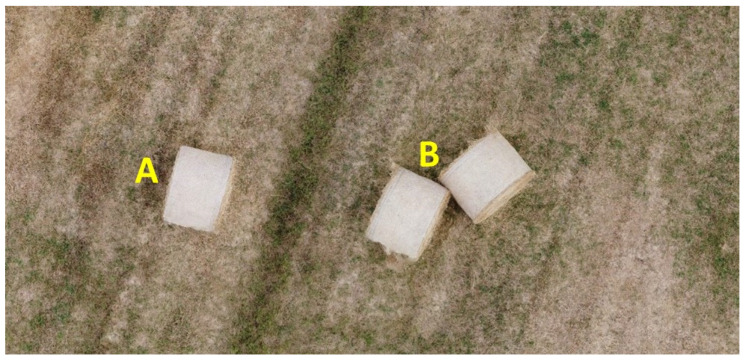
Target objects for the second experiment.

The second experiment was performed in an outdoor environment. It involved simple targets with regular geometry whose volume was analytically measured. In particular, as shown in Figure 7, a field with some hay bales has been surveyed. Their measured volume, assuming the shape to be a cylinder, was 2.41 m^3^. The two of them marked with the letter B are assumed to be unique objects of doubled volume due to their proximity.

The representation provided in Figure 7 is an orthomosaic generated by the fusion of 38 frames acquired by a commercial drone. These data have been used as input for classic SfM processing useful for benchmarking the obtained near real-time estimates.

**Figure 8:**
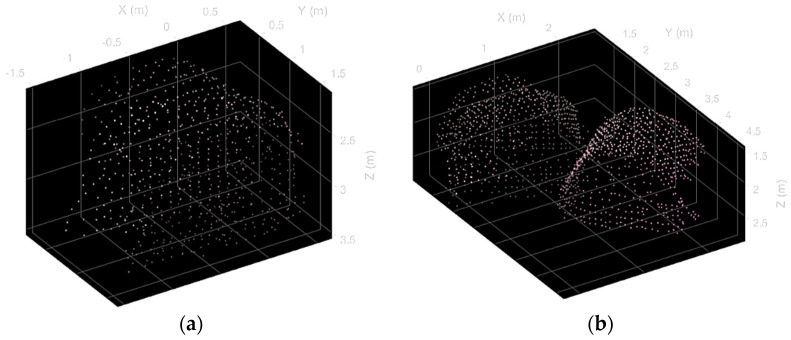
Hay bales reconstruction via direct positioning of depth frames. (**a**) Single hay bales and (**b**) adjacent hay bales.

The three-dimensional reconstruction of the scene objects using the proposed methodology is reported in Figure 8. The grid size has been set to 10 cm. Consequently, the surfaces are reconstructed using a few points, which lowers the quality of the mapping, although there are no visible shape distortions. However, as reported in Table 2, this does not influence the volume estimate’s accuracy. On the other side, the usage of a sparse grid is crucial for computational efficiency, especially in the segmentation phase.

The obtained volume estimates for the single bale and the adjacent bales are 2.49 m^3^ and 5.05 m^3^, respectively, with errors in the order of 3.2% and 4.6%. The estimates obtained exploiting SfM are in the order of 2.66 m^3^ for the single bale and 4.51 m^3^ for the adjacent ones. The errors with respect to measurements are in the order of 9.4% and 6.4%, respectively.

### 3.3. Experiment 3—Construction Site

**Figure 9:**
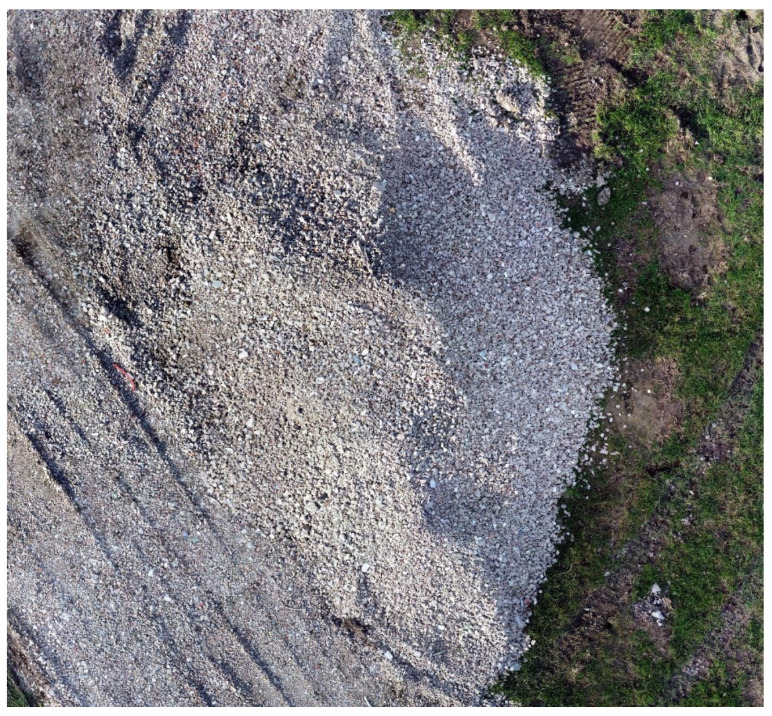
Construction site, orthophoto.

**Figure 10:**
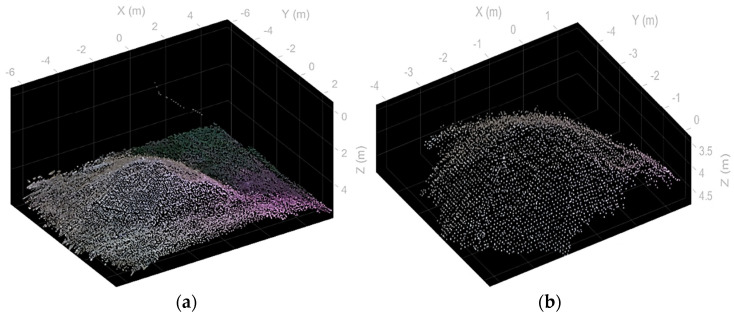
Construction site reconstruction obtained through the direct pose of depth frames. (**a**) Overall scene. (**b**) Segmentation of the stockpile.

This experiment was implemented in a construction site environment and concerned the survey of a gravel pile moved during the excavation for the building of a foundation. The scene is depicted in the orthophoto shown in Figure 9. It has been generated by the photogrammetric fusion of 61 pictures shot by a commercial drone. The three-dimensional reconstruction output by the proposed methodology is shown in Figure 10. In particular, Figure 10a reports the overall scene, while Figure 10b concerns the stockpile isolated from the remaining scene after its segmentation. 

Quantitative data about this experiment are reported in Table 3. It arises that the gap between the photogrammetric estimate and the direct pose one is about 10%. In particular, the volume estimated through photogrammetry is 15.6 m^3^ using a reconstruction grid of about 4 mm. The result obtained from the proposed methodology is 14.2 m^3^ using a grid of 10 cm.

### 3.4. Experiment 4—Micro-Dump

**Figure 11:**
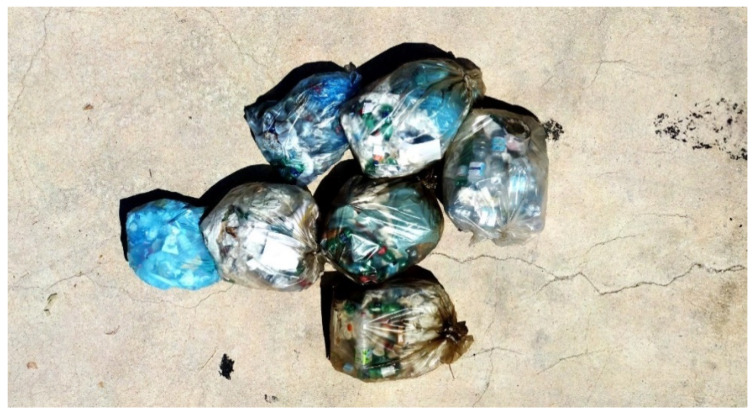
Micro-dump, orthomosaic obtained by fusion of 73 pictures acquired by a commercial drone.

The last experiment deals with the original application for which the system has been designed, i.e., environmental monitoring. In particular, a micro-dump in an operational environment has been surveyed. The target was composed of some waste bags abandoned on the street. Reference data were produced using pictures shot by a commercial drone. They have been fused via photogrammetric processing to create the orthomosaic depicted in Figure 11.

Quantitative data about the experiment are provided in Table 4. It arises that the gap between the photogrammetric estimate and the direct pose one is about 3.5%. In particular, the volume estimated through photogrammetry is 1.51 m^3^ using a reconstruction grid of 1.25 mm. The result obtained from the proposed methodology is 1.46 m^3^ using a grid of 10 cm.

## 4. Discussion

Volume estimation requires three-dimensional modeling of high-resolution data, expert operators, and a significant computational burden necessary to run state-of-the-art SfM techniques. The associated costs are usually due to machines and software licensing needed. As a result, most of the potential users are not attracted by the technology, which could introduce benefits and simplifications in several industrial processes.

In this work, a new cost-effective architecture for near real-time volume estimation has been presented. It tackles some of the bottlenecks in current practices, i.e., the computational burden necessary to implement traditional photogrammetric processes and the connected cost of commercial software, whose performance and usability are significantly higher than (the few) open-source solutions available.

The proposed architecture was principally developed to tackle the problem of illegal dumping and to provide public administrators with a cheap and easy-to-use tool for evaluating the dangerousness of a site. However, the technology’s potential applications are manifold, and the experiments implemented in the validation phase aim to demonstrate its versatility.

The first test was implemented in an indoor GNSS-denied environment and involved small boxes with regular shapes whose analytic volume was available. The output of the proposed methodology and the photogrammetric process were comparable and consistent with the reference value, with an average error of 7% for the direct pose and 25% for photogrammetry. In the evaluation of these numbers, it should be considered that the objects at hand were very small. This means that small deviations from the reference value, even in the order of a few cubic centimeters, can result in a large percentage error. Overall, it is possible to argue that the results obtained with a direct pose of depth frames are comparable with those provided by photogrammetry; nevertheless, the grid used for the reconstruction was three times higher. 

The grid’s density is a parameter that significantly impacts the processing time. The denser the grid, the higher the number of points within the cloud. This results in a higher computational time, especially in the point cloud optimization and segmentation phases. In the above-described experiment, a side objective was to test the mapping capability of the system. Therefore, a rather dense grid has been adopted. This favorably impacted the visual quality of the output at the expense of the computational time in the order of 90 s.

In order to speed up the calculations, the subsequent experiments have been implemented by setting a sparser grid, up to 10 cm. This means that the reconstruction is mainly focused on the envelope of the object, which is sufficient for a reliable volume estimate. This is confirmed by the results of the hay bales experiment. In this case, the visual quality of the reconstruction is much lower than photogrammetry, but the volume estimate is comparable. Overall, the percentage error with respect to the analytic volume is in the order of 4% for the direct pose case and 7.5% for the photogrammetric processing. The reduced density of the reconstruction grid allowed for reducing the computational time up to about 65 s despite the higher number of depth frames processed.

Among the fields that can benefit from the proposed solution, earthworks play an important role due to the continuous moving of goods and terrains that need to be measured. The third experiment had as its objective the estimation of the volume of a stockpile. In this case, as an analytic measurement is not available, the reference value is provided by photogrammetry, as this technique has widely demonstrated its robustness with the application [24,25,26]. As shown in Table 3, the estimate obtained using the proposed methodology is comparable with the photogrammetric one, despite the sparser grid adopted and the reduced number of frames used for the reconstruction. Interestingly this opens it to the possibility of reconstructing the envelope of the surface using few frames and, therefore, few points, thus optimizing computational times, reduced for this experiment up to about 41 s.

The last experiment concerns the original application for which the proposed system has been designed, i.e., the micro-dump’s monitoring. As in the previous experiment, reference data are assumed to be those output by photogrammetry. They are fully comparable with the proposed methodology’s estimate, with the gap between the two values in the order of 3.5% despite the different grid sizes.

Based on the collected data, it is possible to argue that the developed system provides volume estimates fully comparable with those obtainable via SfM. The main limitation is represented by the depth camera’s functioning range, which prevents the survey of objects with heights greater than the range of about 7 m.

## 5. Conclusions

This work presents a new architecture for near real-time volume estimates based on depth cameras. It is composed of a custom flying platform equipped with a payload able to deliver the positioning information needed for placing the depth frames in a three-dimensional reference system. An onboard CPU is responsible for managing the acquisition and running of the developed software for point cloud processing.

The proposed architecture has been extensively tested in the field with experiments concerning different applications such as logistics, agriculture, earthworks, and environmental monitoring. The obtained results have been compared against measurements and state-of-the-art photogrammetry. The benchmark showed that the provided estimates are fully comparable with the reference ones, with average deviations with respect to reference data in the order of 5%.

The main limitation is constituted by the functioning range of the depth camera, preventing the survey of objects having a height greater than 7 m. Inside that range, the proposed architecture provides a quick, cost-effective, and reliable solution to the problem of on-field volumetric estimates.

## Figures and Tables

**Table 1:** Indoor experiment, quantitative data.

Parameter	Direct Pose	Agisoft Metashape
Estimated volume	A = 0.052 m^3^ B = 0.077 m^3^	A = 0.040 m^3^ B = 0.068 m^3^
Error	A = 4% B = 10.5%	A = 23.1% B = 26.5%
Grid size	1 cm	3.27 mm
Average flight velocity	0.16 m/s	
Average flight height	2.67 m	
Number of frames	80	31

**Table 2:** Hay bales experiment, quantitative data.

Parameter	Direct Pose	Agisoft Metashape
Estimated volume	A = 2.49 m^3^ B = 5.05 m^3^	A = 2.66 m^3^ B = 4.51 m^3^
Error	A = 3.2% B = 4.6%	A = 9.4% B = 6.4%
Grid size	10 cm	3 mm
Average flight velocity	0.91 m/s	
Average flight height	5.3 m	
Number of frames	89	38

**Table 3:** Construction site experiment, quantitative data.

Parameter	Direct Pose	Agisoft Metashape
Estimated volume	14.2 m^3^	15.6 m^3^
Gap with reference data	9.8%	
Grid size	10 cm	3.91 mm
Average flight velocity	0.65 m/s	
Average flight height	4.91 m	
Number of frames	43	61

**Table 4:** Micro-dump experiment, quantitative data.

Parameter	Direct Pose	Agisoft Metashape
Estimated volume	1.46 m^3^	1.51 m^3^
Gap with reference data	3.42%	
Grid size	10 cm	1.25 mm
Average flight velocity	0.22 m/s	
Average flight height	3.24 m	
Number of frames	28	73

## Data Availability

Not applicable.
